# The Costs of Evaluating Species Densities and Composition of Snakes to Assess Development Impacts in Amazonia

**DOI:** 10.1371/journal.pone.0105453

**Published:** 2014-08-22

**Authors:** Rafael de Fraga, Adam J. Stow, William E. Magnusson, Albertina P. Lima

**Affiliations:** 1 Instituto Nacional de Pesquisas da Amazônia – Programa de Pós-graduação em Ecologia, Manaus, Amazonas, Brazil; 2 Department of Biological Sciences, Macquarie University, Sydney, New South Wales, Australia; 3 Instituto Nacional de Pesquisas da Amazônia – Coordenação de Biodiversidade, Manaus, Amazonas, Brazil; University of Sydney, Australia

## Abstract

Studies leading to decision-making for environmental licensing often fail to provide accurate estimates of diversity. Measures of snake diversity are regularly obtained to assess development impacts in the rainforests of the Amazon Basin, but this taxonomic group may be subject to poor detection probabilities. Recently, the Brazilian government tried to standardize sampling designs by the implementation of a system (RAPELD) to quantify biological diversity using spatially-standardized sampling units. Consistency in sampling design allows the detection probabilities to be compared among taxa, and sampling effort and associated cost to be evaluated. The cost effectiveness of detecting snakes has received no attention in Amazonia. Here we tested the effects of reducing sampling effort on estimates of species densities and assemblage composition. We identified snakes in seven plot systems, each standardised with 14 plots. The 250 m long centre line of each plot followed an altitudinal contour. Surveys were repeated four times in each plot and detection probabilities were estimated for the 41 species encountered. Reducing the number of observations, or the size of the sampling modules, caused significant loss of information on species densities and local patterns of variation in assemblage composition. We estimated the cost to find a snake as $ 120 U.S., but general linear models indicated the possibility of identifying differences in assemblage composition for half the overall survey costs. Decisions to reduce sampling effort depend on the importance of lost information to target-issues, and may not be the preferred option if there is the potential for identifying individual snake species requiring specific conservation actions. However, in most studies of human disturbance on species assemblages, it is likely to be more cost-effective to focus on other groups of organisms with higher detection probabilities.

## Introduction

Obtaining environmental licenses to build and operate infrastructure or industrial facilities typically requires environmental assessment, and this process usually evaluates abiotic features, such as soil and water, and biotic features, such as fauna and flora. In Brazil, taxonomic groups to be included in environmental-impact assessments are defined by environmental agencies, and these usually include “herpetofauna”, which includes snakes. Compared with other surveyed vertebrate groups, snakes are often rarely encountered, and can be more difficult to detect because they are secretive and cryptic [1], [2]. The problem of detecting snakes may be exacerbated by dense vegetation in rainforest areas, such as those in the Amazon Basin. This raises the question of whether collecting data on snakes is a cost-effective means of evaluating the impacts of environmental change in the rainforests of the Amazon Basin.

Describing human impacts on wildlife assemblages and developing conservation strategies can be based on monitoring that repeatedly measures the biotic response to disturbance [3] and provides data with direct application to setting priorities for research and conservation [4], [5]. Complementary phylogenetic and functional diversities have been shown to be more suitable meaures to assess asemblage changes, in comparison to species densities and composition [6], because disturbance is usually not acting at the level of species alone, but changing a network of abiotic and biotic factors interacting to filter species assemblies [7]. However defining functional groups depends on accurate estimates of multivariate niche overlap, which has been a challenge in tropical forests. Also, phylogenetic diversity alone does not necessarily reflect functional distance, because evolutionary traits may converge and diverge rapidly [6]. Because this study aimed to test only the effects of sampling design on ecological patterns, we represent snake assemblages using the number of species detected per plot system and species composition.

Recently, the Brazilian environmental agencies have recognized the value of standardizing sampling designs, and require or recommend the use of the RAPELD system [8], [9]. RAPELD is an acronym for rapid assessments (RAP) combined with long term ecological research (PELD; in Portuguese). This method was modified from the 0.1 ha survey method developed by Gentry [10], differing primarily in that the direction of the long axis of each plot is along the altitudinal contour; use of different widths of plot for different taxa; and regular distribution of the plots across the landscape to be sampled [8]. Surveying along the contour line reduces the effects of change in altitude along the plot. Altitude probably does not directly affect organisms in lowland Amazonia, but it is related to other factors influencing plant and animal assemblages, such as edaphic characteristics [8].

The RAPELD system was designed to assess ecological parameters, such as species densities and assemblage composition, across spatially standardized sampling units [8]. Compared to individual or species-based sampling, it offers at least four main advantages: (1) the spatialization of sampling units has been useful both for rapid assessments of biological diversity and long-term monitoring; (2) due to its modular design, data from sites with different sampling intensity can be compared; (3) the sample design allows sampling for taxa of different sizes and mobility in the same sampling units; and (4) because the sampling plots follow the altitudinal contours, more precise measures of habitat factors, such as altitude, vegetation and soil characteristics, can be used as predictor variables in ecological models. The RAPELD system has been used to assess ecological and biogeographic processes that generate patterns of animal (e.g. [11]–[13]) and plant (e.g. [14]–[16]) distributions. However, being a relatively recent approach, its application has to be adjusted, especially in relation to the amount of sampling effort required to answer questions relevant to quantifying disturbance from human activities, and also to improve our knowledge about patterns of species distribution at regional scales.

Sampling effort differs among taxa as a function of detectability, and adjustments are important to generate useful results with the least possible financial investment. In fact, high cost has been identified as a major barrier to the maintenance of biodiversity monitoring programs [17], and exceeding the limits of budgets is typical in multi-taxa studies [18], [19]. Recommendations for adjustments of standardized sampling in order to reduce costs have been provided in the Amazon for ants [20], [21] and mites [22], and some high-performance taxa for biological monitoring have been identified [23].

Although monitoring of snakes is mandatory in most impact assessments associated with major infrastructure projects in the Amazon, there has been no evaluation of the cost-effectiveness of targetting snakes. We used data from RAPELD monitoring of snakes in an area of about 1,500 km^2^ covered by primary and secondary tropical rainforest to quantify the loss of information on differences in species densities and composition under reduced sampling effort.

## Materials and Methods

### Study Area

We obtained data on snake species-assemblage composition with the support of the Wildlife Conservation Program from Santo Antonio Energia, the concessionaire responsible for building and operating the Santo Antônio Hydroelectric Plant, in the Madeira River in southwestern Brazilian Amazonia (Rondônia State). This dam, which began operations in 2012, flooded about 210 km^2^ of mainly primary rainforest, but here we use data collected prior to dam construction.

The study area is covered by “terra-firme” (not seasonally flooded) forest, seasonally flooded forests and “campinaranas” (white-sand forests). In the “terra-firme” areas, the canopy is up to 30 m in height and density of the understory varies according to altitude. In areas covered by “campinarana” the canopy is up to 20 m in height, and the understory is rich in ground bromeliads. The flooded forests are restricted to lowland areas near the banks of the Madeira River. Further details on the physiognomic classification can be found in [24].

The climate is consistently warm with average monthly temperatures between 20^o^ and 30°C, and minima and maxima between 18^o^ and 33°C in July. The dry season extends from June to September, with rainfall less than 30 mm per month in June and July, and a rainy season from October to May, with up to 330 mm of rain per month in December and January [25]. Tributaries of varying sizes are present in the study area, and the smallest of these often dry completely in the dry season.

### Sampling design

**Figure 1:**
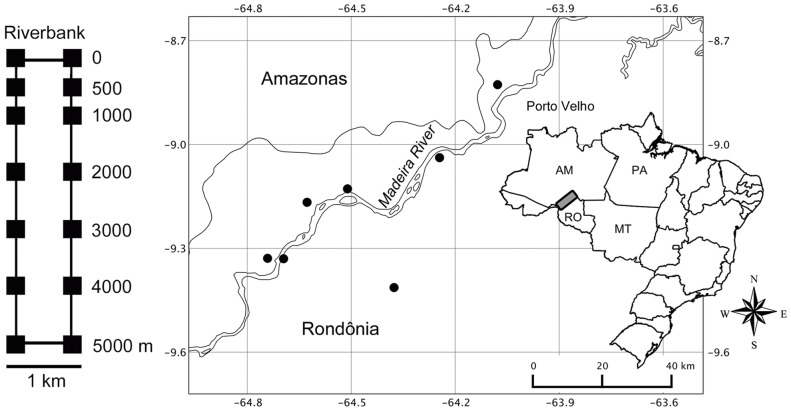
Plot systems in southwestern Amazonia. Sampling systems of 5 km^2^ (black circles) located near the banks of the Madeira River in southwestern Brazilian Amazonia (Rondônia state). In detail on the left side, standard configuration of each system, with 14 plots (black squares).

We sampled seven plot systems (Figure 1), each consisting of two parallel 5 km trails, separated by 1 km. Seven 250 m long by 10 m wide plots with centre lines following the altitudinal contours were installed along each trail (14 per system). Plots were established at distances of 0, 500, 1000, 2000, 3000, 4000 and 5000 m from the river bank. Plot systems are called modules hereafter. All modules were installed perpendicular to the river. Four modules were on the left bank of the Madeira River (Madeira - Purus interfluve), two were on the right bank (Madeira - Tapajós interfluve), and one was on the right bank of the Jaci-Paraná River, a tributary of the right bank of the Madeira River.

### Snake sampling and sampling effort

We found snakes by visually searching at night, limited by space, with two observers per plot. We undertook four sampling campaigns (March-April 2010, November 2010, January 2011 and May-June 2011). Each campaign lasted about 30 days, and all plots were sampled in each campaign. We standardized the search time to one hour per plot (14 hours per module), but we had an average variation of 15 minutes in total time due to differences in the number of snakes encountered. As we did not search for other snakes while processing captured snakes, the effective search time was about one hour in each plot.

We held a maximum of six voucher specimens per species, per module. Voucher specimens were killed by overdose of a topical benzocaine-based anesthesic, which was applied in the oral mucosa. Voucher specimens were fixed by injecting a solution of 10% formalin and they were preserved in a 70% ethanol solution. All voucher specimens were deposited in the herpetology section of the zoological collections of the Instituto Nacional de Pesquisas da Amazônia, Manaus, Amazonas, Brazil. Further details on ethics will be shown below.

### Data analyses

To test spatial autocorrelation among the modules, we used a Moran's correlogram of geographical distance between pairs of modules and Bray-Curtis dissimilarities in species composition between pairs of modules. We used the correlog function of the Pgirmess package [26] in R V.3.1.0.

We used the number of species detected as an index of the species density in modules. The species-accumulation curves based on rarefaction did not approach asymptotes, so we did not attempt to estimate the total number of species vulnerable to our sampling techniques that use each area. Such methods rarely produce useful information for decisions about megadiverse taxa suring short sampling periods [27]. If the methods are not useful to detect differences in observed species density, they are unlikely to be useful to compare estimates of total number of species, which have far greater standard errors. It was not necessary to use rarefaction to account for differences in search effort between modules because the same temporal and spatial efforts were expended in each module.

**Figure 2:**
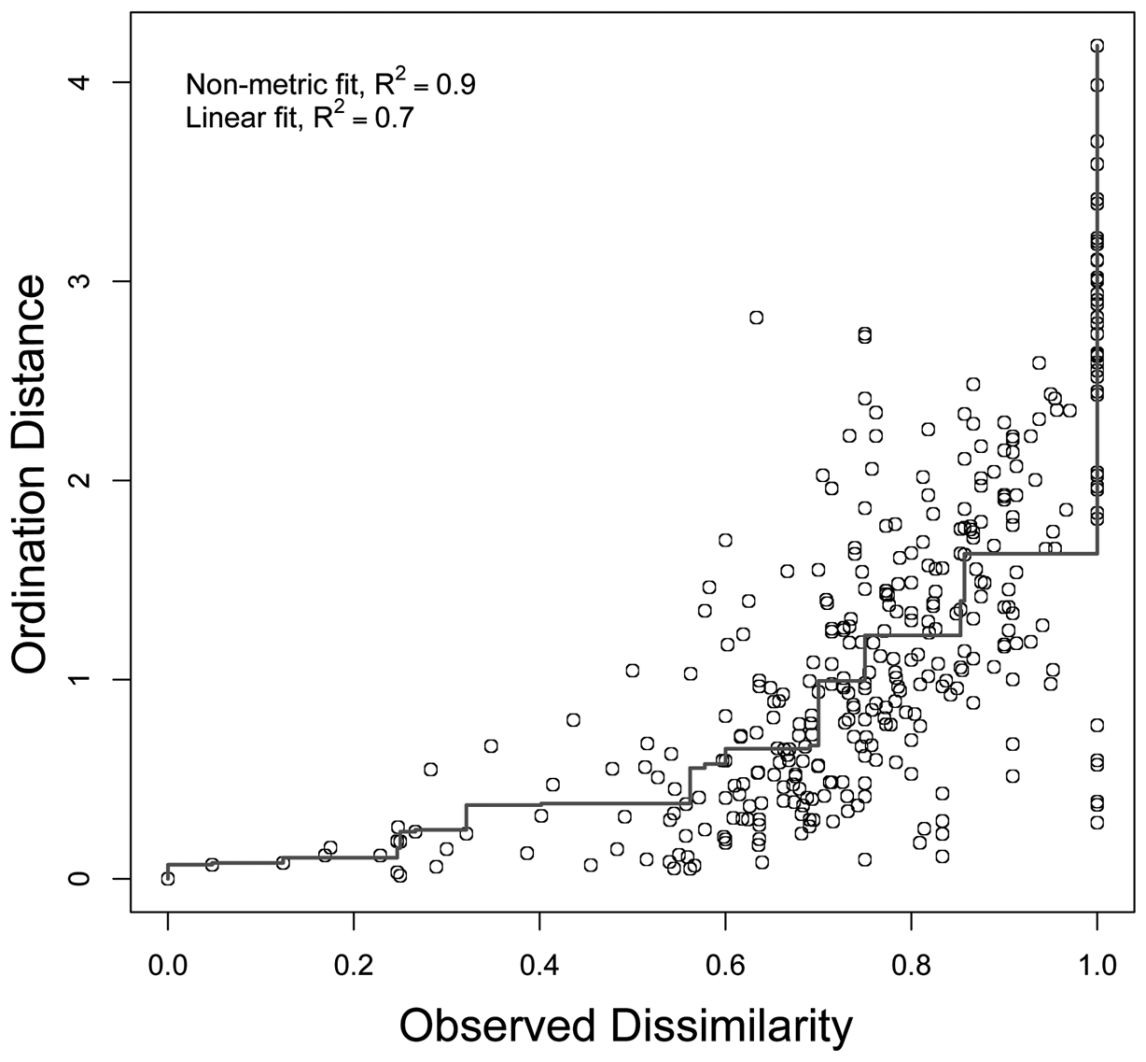
Shepard diagram. Relationship between NMDS ordination distance and original observed distance. NMDS ordination was undertaken on an abundance per species matrix.

We evaluated variation in assemblage composition using a dissimilarity matrix among modules. Dissimilarities were calculated by applying the Bray-Curtis index to the number of individuals per species, per module. We were interested in obtaining one-dimensional proportions of multivariate Bray-Curtis dissimilarities among modules, in order to use t-tests and general linear models. We reduced the dimensionality of the dissimilarity matrix and reordered the modules using Non-Metric Multidimensional Scaling (NMDS) in one dimension. We have chosen this method rather than alternative ordinations such as PCoA, because NMDS is apparently less sensitive to arch effects generated by heterogeneity in species distributions [28]. Moreover, NMDS has been recognized as the most efficient method to recover original multivariate ecological distances [29]. However, we also ran the analyses using PCoA ordinations and they produced qualitatively similar results not reported here. We used the metaMDS function (arguments k = 1, distance = “bray”, trymax = 1000) of the Vegan package [30] in R v2.15.2. The NMDS axis captured 60% (P<0.0001) of the variation in species composition among plots (Stress = 0.15). The reduction in dimensionality often causes distortion in relation to the observed dissimilarities [31], although distance can be reduced by rearranging the placement of points along the NMDS axis [32]. We reordered 1,000 times and we used the Shepard diagram drawn by the function stressplot in the Vegan Package in R to show that the observed dissimilarities and the ordination distances were up to 90% correlated (P<0.0001, Figure 2).

We used paired t-tests to investigate the differences among three different sampling intensities on the number of species recorded and on assemblage composition (NMDS scores). In the first test, we used complete modules (5 km^2^) to show the changes in number of species and assemblage composition due to increasing the number of observations (campaigns). In the second test, we used the maximum number of observations to pair complete modules with modules constituted by only one 5 km^2^ trail (1.25 km^2^). In the third test, we used the maximum number of observations to pair complete modules with modules constituted by two 3 km trails (3 km^2^ modules). The NMDS ordination produced some negative scores, and they directed the vectors (modules) in opposite directions in paired t-tests, canceling each other. Therefore we added 1 to the NMDS scores to avoid negative numbers. To quantify the similarity of the representation of assemblage composition among modules with different sizes and sampling efforts, we tested for associations among NMDS scores using general linear models.

The major rivers in the Amazon basin are associated with the limits of species distributions of several taxonomic groups (e.g. [33]). Although rivers have not been identified as resulting in vicariance for snakes, we compared number of species per module and assemblage composition on the opposing river banks of the Madeira River using an analysis of similiarities (ANOSIM) with assemblage composition represented by NMDS scores. We used the function anosim of the Vegan package in R.

### Detection probabilities

We quantified detection probabilities for each species using single-season models based on presence-absence data in the Program Presence v.5.2, with 100 bootstrap randomizations [34]. A single-season model provides probabilities of occupancy when detection of the target species is not guaranteed, even in places where they are present. The estimated occupancy and detection probabilities describe a history of detecting species over a series of surveys in the same locations [34]. Although the Amazon rainforest is apparently homogeneous on satellite images, subtle changes in habitat features across the landscape at a scale of a few kilometers can influence co-occurrence of species in some taxonomic groups, such as frogs [13], understory birds [12] and snakes [11]. Detection probabilities possibly vary slightly among areas within each module as a function of change in habitat features, such as vegetation density along the trails. We expected higher detectability in more open plots and the number of trees was quantified for all plots during the impact assessment for the hydroelectric dam, but differences in the number of trees among modules were negligible (ANOVA F_6-82_ = 1.836, P = 0.1). Other environmental factors, such as distance from the streams, can directly affect the composition of snake species in Amazonia [11]. However most of the species recorded here are widely distributed in the study area, and similarity in assemblage composition of up to 60% among modules is expected at scales of tens of kilometers (see [35]). Therefore, we expected the occurrence of all species in all modules, with differences in co-occurrence resulting from the variation in habitat use over a few kilometers. As we were interested in estimating average detectability per species scaled to tens of kilometers, we assumed the same probabilities of occupancy and detection across all modules, and across all campaigns. The average detectability, rather than detectability on any given occasion is what is needed to compare costs of detection of different species in general surveys.

### Cost estimates

To estimate the cost of sampling snakes, we considered fuel for transport among modules, batteries for headlamps, food and field-assistant salaries. We did not include the costs of construction and maintenance of the modules because the same field infrastructure was used for sampling many other taxa. For fuel, we calculated the cost considering the average consumption per km for a diesel-powered pickup truck for modules accessible by road, and the average consumption per kilometer of a boat powered by a 60 hp gasoline outboard motor for modules accessible by river. To estimate the number of headlight batteries, we considered eight people searching for snakes simultaneously (two per plot), each carrying a headlamp powered by three AA batteries. The batteries were changed every second night. For food, we used $ 8.68 United States Dollars (USD) per day, per person. This is an average value on the local market. For payment of field assistants, we used a daily value of $ 21.71 USD, a work-contract stipulated value.

### Ethics and data availability

Snakes were collected under IBAMA/SISBIO (Ministry of Environment, Government of Brazil) permit n^o^ 02001.000508/2008-99. This permit was subject to approval of all procedures for catching and collecting snakes, and it was allowing us to collect eight specimens per species, per module. However the limit has not been reached for any of the species found.

All data are available for free download on the website of the Programa de Pesquisa em Biodiversidade (PPBio) http://ppbio.inpa.gov.br/knb/metacat?action=read&qformat=ppbio&sessionid=0&docid=naman.594.1

## Results

### Species densities and assemblage composition

We found 41 species of snakes (Table 1), but the number of species included in each test depended on the module size and number of observations. Neither the variation in number of species detected nor the assemblage composition among modules was spatially autocorrelated (P>0.25 in all cases). The number of species detected per module varied from nine to 21, and did not differ when the opposite banks of the Madeira River were compared (ANOVA F_1-6_ = 0.762, P = 0.83) and assemblage composition based on NMDS scores was only 4% (P = 0.01) different when comparing either side of the river.

**Table 1:** Snake species found in seven 5 km^2^ sampling systems in the southwestern Brazilian Amazon.

Taxon	N	O.M.	P
**Boidae**			
*Boa constrictor* Linnaeus, 1758	4	43	0.18 (0.03–0.63)
*Corallus batesi* (Gray, 1860)	2	28.6	0.07 (0.01–0.24)
*Corallus hortulanus* (Linnaeus, 1758)	10	57.1	0.3 (0.11–0.6)
*Eunectes murinus* (Linnaeus, 1758)	1	14.3	0.03 (0.005–0.21)
**Colubridae (Colubrinae)**			
*Chironius fuscus* (Linnaeus, 1758)	1	14.3	0.03 (0.005–0.21)
*Chironius multiventris* Schmidt & Walker, 1943	3	42.8	0.1 (0.03-0.28)
*Dendrophidion dendrophis* (Schlegel, 1837)	1	14.3	0.03 (0.005–0.21)
*Drymoluber dichrous* (Peters, 1863)	7	42.8	0.22 (0.0009–0.63)
*Mastigodryas boddaerti* (Sentzen, 1796)	2	14.3	0.07 (0.01–0.24)
*Oxybelis aeneus* (Wagler, 1824)	3	28.6	0.1 (0.03–0.28)
*Pseustes poecilonotus* (Günther, 1858)	2	28.6	0.07 (0.01–0.24)
*Pseustes sulphureus* (Wagler, 1824)	1	14.3	0.03 (0.005–0.21)
*Rhinobothryum lentiginosum* (Scopoli, 1785)	4	42.8	0.18 (0.03–0.63)
*Spilotes pullatus* (Linnaeus, 1758)	1	14.3	0.03 (0.005–0.21)
**Colubridae (Dipsadinae)**			
*Apostolepis nigrolineata* (Peters, 1896)	1	14.3	0.03 (0.005–0.21)
*Dipsas catesbyi* (Sentzen, 1796)	11	85.7	0.32 (0.17–0.51)
*Dipsas indica* Laurenti, 1768	3	42.8	0.1 (0.03–0.28)
*Drepanoides anomalus* (Jan, 1863)	4	42.8	0.18 (0.03–0.63)
*Helicops angulatus* (Linnaeus, 1758)	2	28.6	0.07 (0.01–0.24)
*Imantodes cenchoa* (Linnaeus, 1758)	9	71.4	0.3 (0.11–0.6)
*Leptodeira annulata* (Linnaeus, 1758)	19	100	0.42 (0.26–0.61)
*Liophis reginae* (Linnaeus, 1758)	2	28.6	0.07 (0.01–0.24)
*Liophis typhlus* (Linnaeus, 1758)	2	28.6	0.07 (0.01-0.24)
*Oxyrhopus melanogenys* (Tschudi, 1845)	5	71.4	0.19 (0.07–0.36)
*Oxyrhopus occipitalis* (Wied-Neuwied, 1824)	2	14.3	0.07 (0.01–0.24)
*Oxyrhopus petolarius* (Linnaeus, 1758)	1	14.3	0.03 (0.005–0.21)
*Philodryas argentea* (Daudin, 1803)	6	42.8	0.19 (0.07–0.36)
*Philodryas georgeboulengeri* (Grazziotin et al., 2012)	13	57.1	0.36 (0.14–0.66)
*Pseudoboa coronata* Schneider, 1801	2	28.6	0.07 (0.01–0.24)
*Pseudoboa martinsi* Zaher, Oliveira & Franco, 2008	1	14.3	0.03 (0.005–0.21)
*Siphlophis compressus* (Daudin, 1803)	10	71.4	0.3 (0.11–0.6)
*Siphlophis worontzowi* (Prado, 1940)	2	28.6	0.07 (0.01–0.24)
*Taeniophallus* sp.	5	57.1	0.19 (0.07–0.36)
*Thamnodynastes pallidus* (Linnaeus, 1758)	1	14.3	0.03 (0.005–0.21)
*Xenopholis scalaris* (Wucherer, 1861)	5	42.8	0.19 (0.07–0.36)
**Elapidae**			
*Micrurus hemprichii* (Jan, 1858)	4	42.8	0.18 (0.03–0.63)
*Micrurus lemniscatus* (Linnaeus, 1758)	5	42.8	0.19 (0.07–0.36)
*Micrurus remotus* Roze, 1987	4	28.6	0.18 (0.03–0.63)
*Micrurus surinamensis* (Cuvier, 1817)	1	14.3	0.03 (0.005–0.21)
**Viperidae**			
*Bothrops atrox* (Linnaeus, 1758)	18	85.7	0.39 (0.2–0.5)
*Bothrops bilineatus smaragdinus* Hoge, 1966	1	14.3	0.03 (0.005–0.21)

N = Number of individuals recorded in the whole study, O.M. = Proportion of modules estimated to be occupied (%), P = species detection probability and confidence intervals (95%) for a single survey of a module.

**Figure 3:**
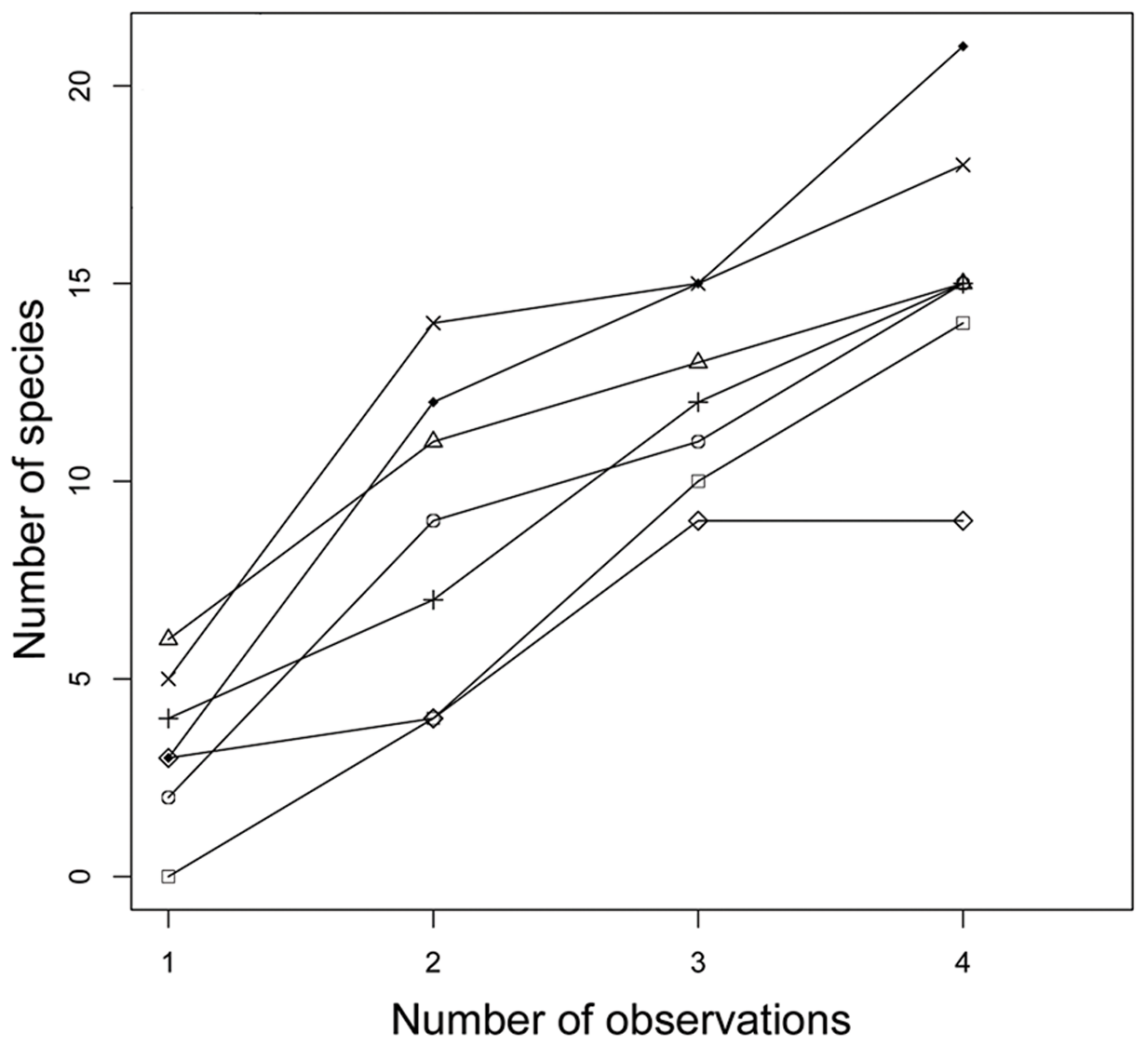
Cumulative number of snake species. Cumulative number of snake species in standardized modules in southwestern Brazilian Amazonia. Modules were sampled 4 times. Different symbols represent different modules.

**Figure 4:**
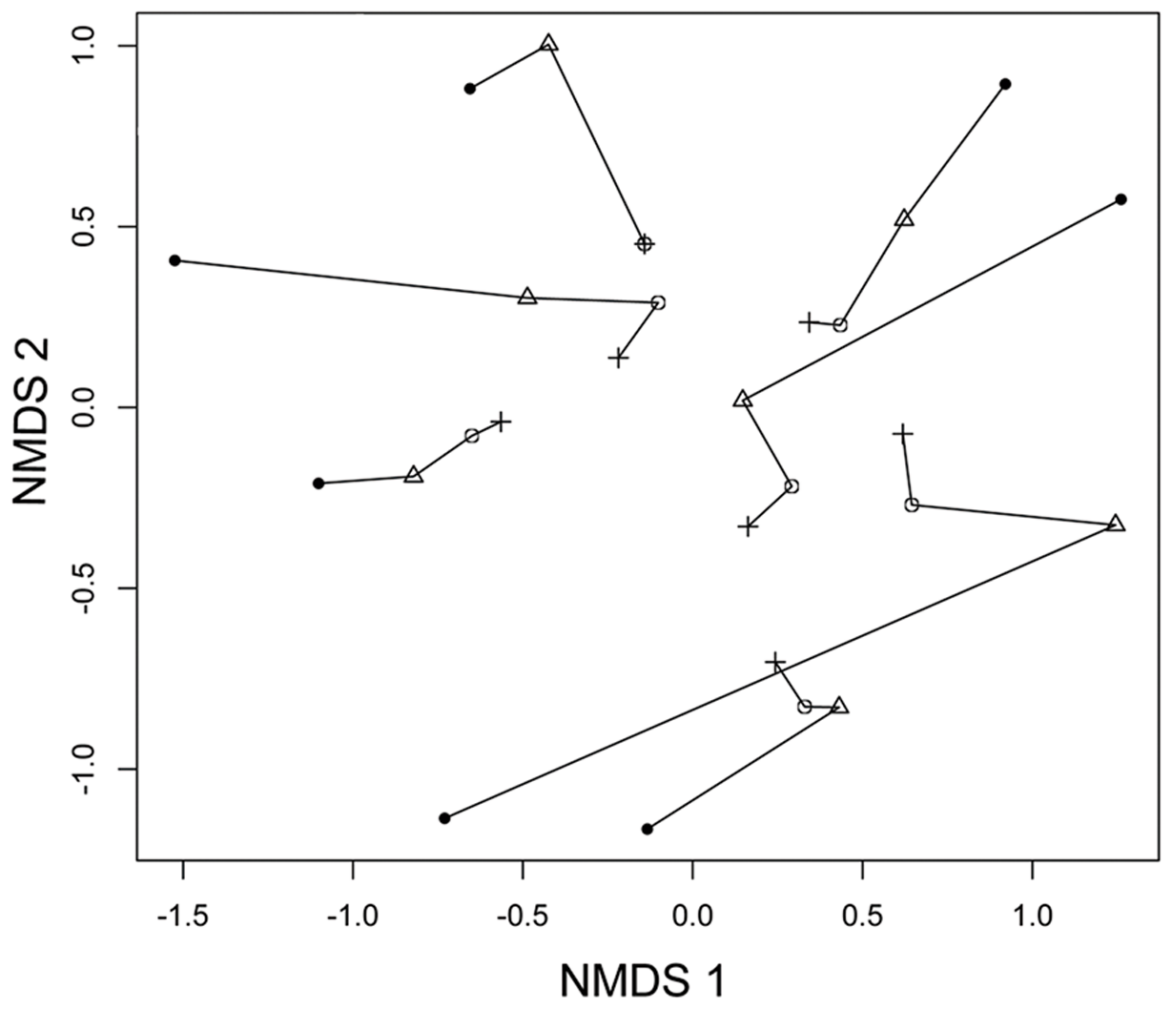
Representation of snake assemblage. Multivariate representation of variation in snake assemblages among modules, based on NMDS scores. Lines connect data for the same module based on different levels of sampling. Black circles  =  one observation, triangles  =  two observations, open circles  =  three observations and crosses  =  four observations.

Using complete modules, species density increased with the number of observations (Figure 3), even increasing between the third and fourth sampling occassions (t_1-6_ = 4.459, P = 0.004). The assemblage composition (Figure 4) differed depending on the number of sampling occassions per module (t_1-6_ = 2.497, P = 0.04). However, a general linear model indicated that NMDS scores were correlated between two and three observations (r^2^ = 0.85, P = 0.001), and highly correlated between three and four observations (r^2^ = 0.97, P = 0.00001). The assemblage composition resulting from four surveys per module was based on more species but provided a representation similar to that with only two surveys per module.

**Figure 5:**
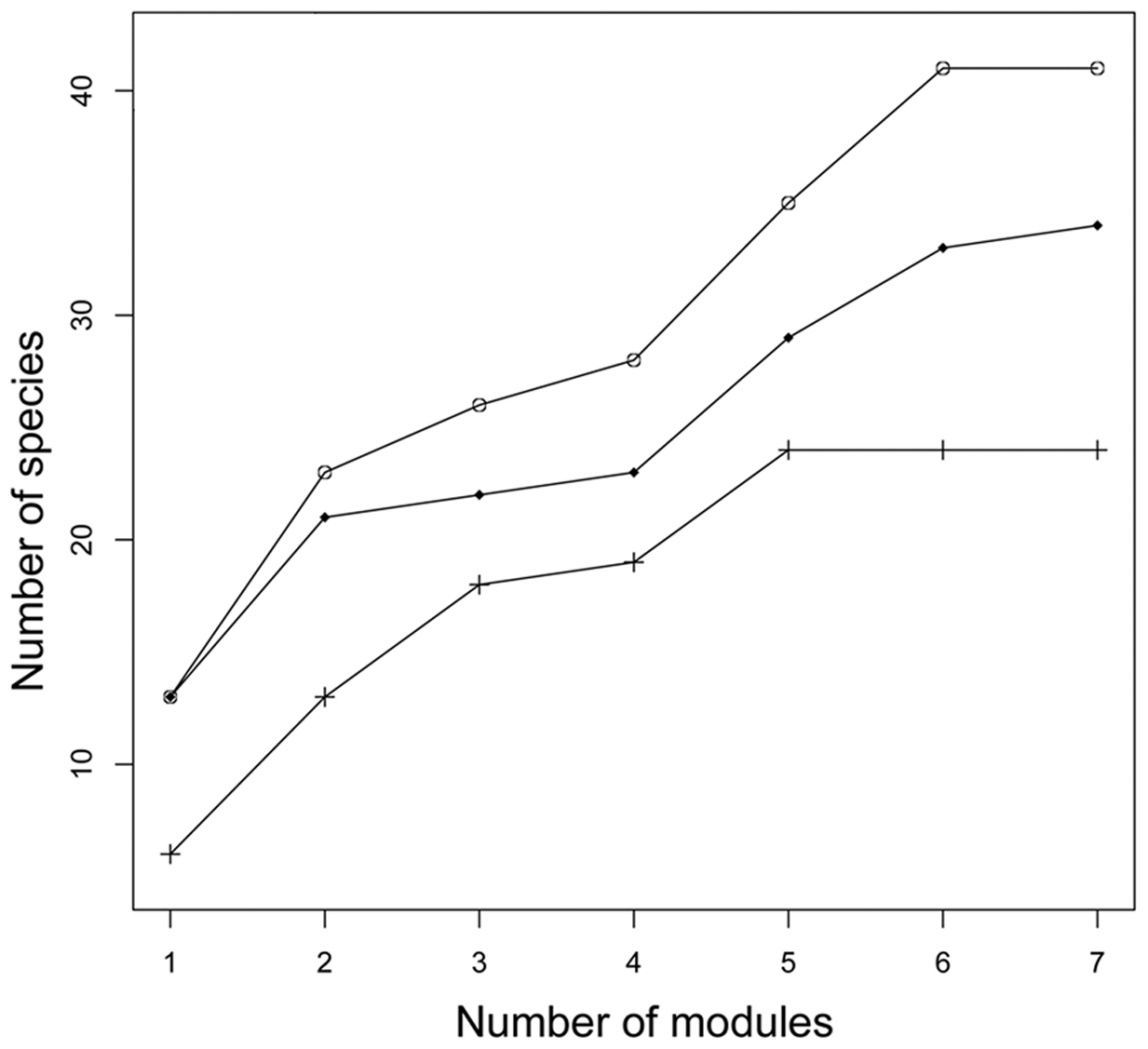
Number of snake species per sample design. Cumulative number of snake species with increasing number of standardized sample modules surveyed in southwestern Brazilian Amazon. Open circles  =  modules with two 5 km trails (5 km^2^), diamonds  =  modules with one 5 km trail (1.25 km^2^) and crosses  =  modules with two 3 km trails (3 km^2^).

**Figure 6:**
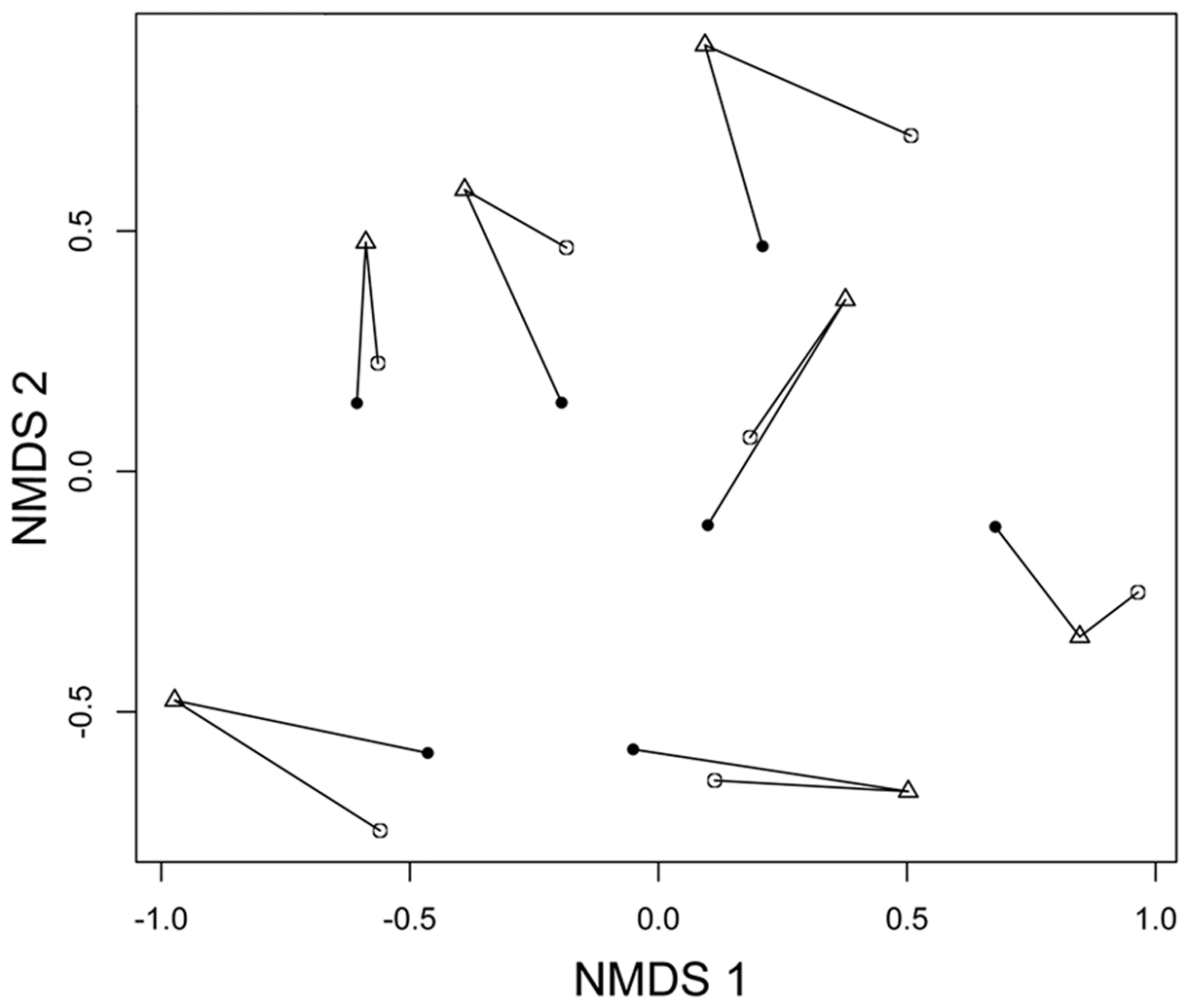
Representation of snake assemblage per sample design. Multivariate representation of variation in snake-assemblage composition based on NMDS scores from data obtained in standardized sampling modules in southwestern Brazilian Amazonia. Lines connect the same module sampled at different intensities. Black circles  =  modules with two 5 km trails (5 km^2^), triangles  =  modules with one 5 km trail (1.25 km^2^) and open circles  =  modules with two 3 km trails (3 km^2^).

Removal of one trail per module (1.25 km^2^ modules) significantly reduced (t_1-6_ = 7.262, P = 0.0003) the number of species encountered (Figure 5). Assemblage composition (Figure 6) also differed between complete and partial sampling of modules (t_1-6_ = 2.404, P = 0.05), but NMDS scores for complete and partial sampling were correlated in a general linear model (r^2^ = 0.62, P = 0.02). Reduction of 2 km in each module (3 km^2^) significantly reduced the number of species detected (t_1-6_ = 4.289, P = 0.005). However, assemblage composition did not differ significantly (t_1-6_ = 1.347, P = 0.21), and NMDS scores were correlated (r^2^ = 0.48, P = 0.04). Although the size of the modules influenced the number of species and assemblage composition, conclusions based on similarity in assemblage composition among modules were similar with up to a 50% reduction in the size of the modules.

### Detection probabilities

**Figure 7:**
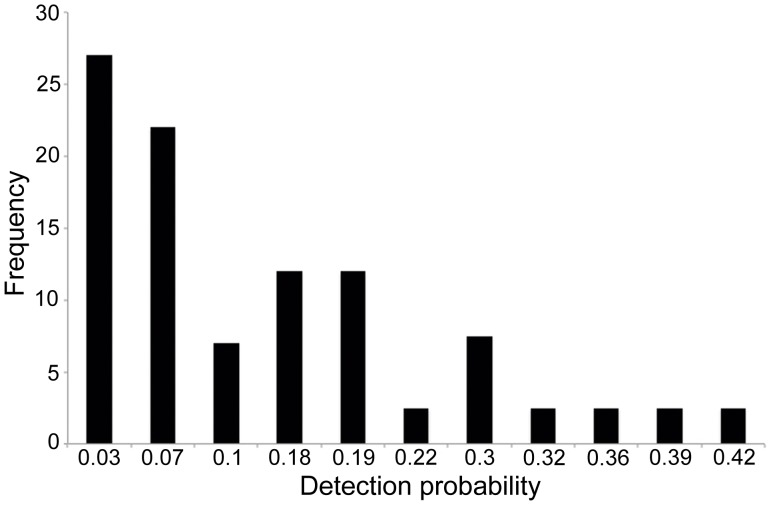
Detection probabilities of Amazonian snakes. Frequency of species of snakes with different probabilities of detection in visual surveys of RAPELD modules.

Detection probabilities of species per expedition per module ranged between 0.03 (SE = 0.03) and 0.42 (SE = 0.09), and were below 10% for almost half of the species detected (Figure 7). Confidence intervals for detection probabilities were very wide for most species (Table 1).

### Costs

Each full survey of all modules cost $ 5,450 USD, and the full study cost $ 21,799 USD (Table 2). The highest costs were for field assistants, followed by food, headlamp batteries and fuel. We had an encounter rate of 0.89 snakes per hour (total number of individuals/total search time). Considering only the costs of food, field assistants, headlamp batteries and fuel, we calculated the cost to find a snake as $ 120 USD (total cost/total number of snakes found).

**Table 2:** Costs for sampling snakes in standardized modules in southwestern Brazilian Amazon.

	Each survey	Full study
Field assistances	2,605.00	10,420.00
Food	2,084.00	8,336.00
Batteries	625.00	2,500.00
Fuel	136.00	544.00
**Total per observation**	**5,450.00**	
**Total for the full study**		**21,800.00**

The values are in United States Dollars.

## Discussion

### Sample reduction and decision-making

The number of species per module increased with each additional survey, and there was no tendency for the rate of species accumulation to lessen with the maximum sampling effort. About 95 snake species occur in the region of Porto Velho, state of Rondônia (literature compilation in [36]), more than twice the number of species found in this study. However, this is an estimate based on decades of herpetological collection, an effort generally not viable for biological monitoring applied to assess the impact of human disturbance. Impacts of human activities have been assessed using secondary data, but this method is not appropriate for detecting the influence of habitat factors on the regional distribution of species, because these data usually are based on specimens, and not sites, as sampling units. In addition, secondary data on snakes are generally not comparable due to the lack of sampling standardization and the sampling of snakes in this manner is rare in Brazil (for exceptions, see [11], [37], [38]).

Although there was variation in assemblage composition with increasing number of observations, the general linear models indicated that two surveys were sufficient for multivariate techniques to capture patterns of species dissimilarities between 5 km^2^ modules. Removal of an entire trail from each module (1.25 km^2^ modules) caused differences in the assemblage composition between pairs of modules, but not in a general linear model. Similar multivariate patterns were detected even with reduced sampling effort in time and space. However, paired tests are strongly influenced by the spatial configuration of the sampling units and the number of observations and this has traditionally varied among studies. The sampling configuration has often varied in relation to the answers required for the land-use management or improving knowledge about regional patterns of species distribution. A standardized sampling system overcomes some of these issues and allows data to be compared among studies.

We found that assemblage composition varied by up to 40% among modules. Differences in assemblage composition should be expected as a response to environmental gradients that subtly change on a scale of a few kilometers (e.g. [11]). We could have found the same pattern of differences in assemblage composition with a 50% reduction in the costs of food, fuel for transport, batteries for headlamps and field assistants, or a 40% reduction in construction costs of the modules (by reducing of 2 km on each trail). However, these decisions would imply the loss of about 24% of the detected species, and make it difficult to detect local variations in assemblage composition. Deciding on sampling effort should be guided by the target-issues, but such decisions are usually made subjectively by the researchers, rather than in consultation with the regulatory agencies that will make management decisions [39].

### Species detectability

All of the tests were limited by the low detectability of most species and associated wide confidence intervals, despite the relatively high sampling effort in time (980 observers*hours of searching) and space (3,500 ha of plots). We can not discount an effect of variation in the probability of detection of species among the modules because different types of habitat occur in patches throughout each module. However, we do not expect much more than the maximum detectability found in this study (42%), because detection probability for snakes have generally been found to be below 40%, even in areas known to be occupied (e.g. [37], [40]), and species that are not considered rare can be virtually undetectable, showing detection probabilities below 1% [2]. Failure to detect species in occupied habitats can generate erroneous predictions of species responses to natural variation in habitat factors [41], and generating reliable models of habitat use depends on very high sampling effort, and consequently very high financial costs. Thus, conservation programs based on species that are difficult to detect usually prioritize areas where habitat factors favor the detection, and not necessarily the responses of organisms to habitat change [42]. Overcoming these biases for snakes would come at a high monetary cost. In assessments of disturbance, it is generally advantageous to focus on sampling a limited set of high-detectability taxa that reflect the broader patterns of diversity [43], [44].

### Cost-benefit

We calculated the cost to find a snake as $ 120 USD. Mesquita et al. [45] spent $ 0.49 USD per snake found by visual search in a semiarid region of Brazil, more than 230 times cheaper than this study. Differences in costs should be more evident among regions with different climate, terrain, vegetation and logistics, but those authors did not present a detailed description of the spatial distribution of the sites observed, and therefore the independence of sampling units was not clear. Furthermore, they did not estimate costs for food, fuel and field assistants, without which data collection would be impossible in the wide-scale sampling used in this study.

Although snakes are highly diverse in species and lifestyles in the Amazon, ecological models based on snake data are frequently influenced by false absences, resulting from low detectability and lack of specific methods for efficiently catching specimens (see [46]). Complementary methods for detecting snakes, such as passive traps, can increase species lists, especially because they optimize the catching of small litter and fossorial snakes (e.g. [47], [48], but the results usually do not justify the high cost and physical effort (e.g. [45]).

In some cases, a snake species with a limited distribution may be the primary target for impact assessment (e.g. *Bothrops alcatraz* and *Bothrops insularis*, species endemic to islands in southeastern Brazil), and general snake sampling during environmental-impact studies may be useful to generate landscape-scale models of species distribution and to obtain natural-history data, such as those on diet and reproduction. Futhermore, snakes can be found during visual search for other taxa, such as lizards and frogs. However, our simulations show that snakes are generally not good models for impact assessments, because detection of strong ecological patterns requires high financial costs, and cost reduction depends on subjective decisions about the importance of the lost information for management decisions. Also, if visual identification is not sufficient, collecting snakes may involve risks to personnel, and such risks might not be covered under general work insurance.

Limiting studies on human disturbance to a few taxa may be a necessary pragmatic decision in biodiverse regions, because weak cross-taxon congruence in assemblage composition is expected among higher-taxa [49]. Different groups of organisms may respond to habitat changes in different ways, and thus represent only small fractions of the total ecological funcionality of an area (see [50], [23]). Therefore we do not expect that there are suitable ecological surrogates for snakes, which makes it a difficult decision to not include them in multi-taxa studies aiming to understand human disturbance on the overall functioning of biodiversity networks. Combining snake sampling with surveys on other taxa, such as frogs and lizards, could potentially add value to measurements of biological diversity. However, due to the very low probability of detection, the data may not be useful for impact assessments. In any case, no snake species or assemblages are considered endangered in the Brazilian Amazon [51]. Therefore, due to the practical restrictions imposed by limitations of time and money, we recommend that reports on environmental impacts in the Amazon should focus primarily on high-detectability taxa, for which these limitations usually are less of a barrier for decision-making. Strategically appropriate taxa are those that simultaneously reflect useful measures of ecological patterns and are feasibly sampled [23], such as birds [52] and dung beetles [53]. We do not have detailed detectability probabilities for these species. However, standard methods obviously collect more than the mean of 6.5 individuals and 5.75 species of snakes per module per sampling occasion that we found, so it can be expected that they will cost much less than the U.S. $120 per individual that we found for snakes. If the number of taxa is reduced, the resources saved can be used to increase the spatial scale of sampling, which is usually one of the most important restrictions on decision making in the context of environmental impacts [9].
